# The Clothing of Children

**Published:** 1907-03-09

**Authors:** 


### The Clothing of Children.

The vitality of old traditions and beliefs, and also the mischief they may do, find no better illustrations than may be seen in the up-bringing of children. The most serious instances are met with in regard to infant feeding, especially in the general idea that milk alone is not a sufficient food, an idea which only too often finds practical expression in the horrible custom of giving a new-born infant butter and sugar, to be followed probably a little later by some much-advertised starchy food. Whilst, however, the mischief wrought by improper feeding is the more serious, the evil consequences of old superstitions in regard to the clothing of children are hardly less wide-spread. Especially may be mentioned such deep rooted errors as that children need hardening by exposure to cold; and that if the chest is adequately protected the rest of the body, and the extremities, may be left to take care of themselves. In regard to the first point, we need to impress upon mothers the all-important difference between exposure of a child to cold air when properly and when improperly clad. Certainly after the first few weeks of life a healthy baby may, and in fact should, be taken out in all weathers in which a nurse can go out, provided always that in cold weather the infant is thoroughly wrapped up. The same is true in the case of a child old enough to run about, if only, should the clothes get at all damp either from rain or perspiration, they are changed immediately. Whilst under proper conditions exposure to cold can do nothing but good, it must be remembered that, owing to the relatively larger area of cutaneous surface to body weight in a child as compared with an adult, the former loses heat more rapidly than the latter, and is therefore more susceptible to cold. Consequently the hardening process as commonly practised is apt to be more or less equivalent to the Spartan custom of exposing delicate children, its only merit probably being that it helps in some degree to ensure the survival of the fittest, although Nature does this efficiently enough without our assistance.

The other notion already mentioned is that the chief object in clothing a child is to protect the chest, and that if this is done it matters but little how scanty is the covering to other parts of the body, although, curiously enough, mothers who dress their children in this way often reverse the procedure for themselves, by going out in the evening with practically bare chests: such are the vagaries to which fashion leads! It is no uncommon thing to see a child with its legs bare and the abdomen quite insufficiently protected, whilst the chest is enveloped in layer upon layer of different articles of apparel. This unequal distribution of the clothes is largely, no doubt, a result of the belief that bronchitis is especially due to cold acting directly on the chest-walls. Two facts are overlooked; one is that cold acting on parts of the body other than the chest may quite well give rise to bronchitis; the other, that chills are responsible, particularly in childhood, for numerous evils besides respiratory troubles. Notably is this the case in regard to digestive disturbances, which in many children are quite as readily set up by a chill as by improper food. A child needs a uniform, but not very thick, layer of flannel next the skin, not only of the chest, but also of the abdomen and legs. Infants should wear flannel or woollen drawers over the napkin, and from the age of about two years flannel combinations are desirable. In addition, save perhaps in the height of summer, long stockings should be worn, reaching above the knees so as to meet the combinations, or else gaiters should be put on the legs each time the child goes out; at the same time the garments on the upper part of the trunk may often be reduced. In this way the same weight of clothing as before, properly distributed over the body, will ensure adequate protection to the legs and abdomen, and will obviate the serious risks which arise from chill to these parts.

Only too often, despite all that a medical man can say, ignorant mothers, and such ignorance is far from being confined to any special social class, prefer to follow the dictates of fashion or the advice of some neighbour who has reared, or more often buried, a large family of children, or of some self-opinionated old nurse full of ancient prejudices and superstitions. The doctor is looked upon as a faddist or as not practically acquainted with the management and up-bringing of children. All that he can do is to preach, in season and out of season, that children should be clad on the same lines as adults, but with even greater care, whilst nurses who have received a modern training should be able in ever-increasing numbers to support his efforts.

